# High-SNR Whole-Brain Mesoscale Diffusion MRI Using Rotating-View Acquisitions (ROVER-dMRI)

**DOI:** 10.64898/2026.09.25.754394

**Published:** 2026-10-01

**Authors:** Qiang Liu, Yunqi Wang, Yiang Pan, Xingwang Yong, Yuting Chen, Hwihun Jeong, Nan Wang, Erpeng Dai, Jarrett Rushmore, Carl-Fredrik Westin, Lauren J. O’Donnell, Berkin Bilgic, Kawin Setsompop, Deniz Erdogmus, Lipeng Ning, Yogesh Rathi

## Abstract

**Purpose:**

We aim to acquire and reconstruct high signal-to-noise ratio (SNR), whole-brain, mesoscale (isotropic submillimeter) diffusion MRI (dMRI) in vivo in a clinically feasible scan time.

**Methods:**

We present ROVER-dMRI, a unified rotating-view acquisition and super-resolution reconstruction framework. Thick slices (up to 10 mm) acquired across many rotated orientations improve the base SNR, and a shared multi-resolution hash-encoded implicit neural representation (INR) with per-direction MLP head and a physics-informed, Bloch-simulated slice-profile forward model reconstructs these views into a continuous isotropic volume (up to 13× super-resolution) in a self-supervised manner. ROVER-dMRI was evaluated in vivo on human brain (0.78 mm isotropic and 0.5 mm isotropic) and monkey brain (0.6 mm isotropic) across MRI platforms with varying gradient performance, and benchmarked against gSlider-SMS and least-squares super-resolution reconstruction (LS-SRR).

**Results:**

At 0.78 mm isotropic resolution, ROVER-dMRI diffusion-weighted images achieved higher SNR than the vendor product sequence, whose images were limited by the noise floor, while its directionally encoded color (DEC) maps showed lower noise than gSlider. When reducing the number of views from 20 to 13 (reducing the scan time by 65%), ROVER-dMRI acquisition/reconstruction preserved all fine details in the fiber orientations. In the monkey brain, its DEC map matched a 60-direction gSlider acquisition at only 40% of the scan time. ROVER-dMRI preserved coherent fiber-orientation-distribution (FOD) structure, including crossing fibers, and recovered fine details in the cerebellum at 0.5 mm isotropic, that are not visible at 0.78 mm resolution. Compared to state-of-the-art 0.5 mm methods, ROVER-dMRI reduced scan time by 73% while acquiring more diffusion directions.

**Conclusion:**

ROVER-dMRI offers a practical route to high-SNR whole-brain mesoscale diffusion imaging in vivo, with the pulse sequence and reconstruction pipeline released as open source.

## Full Text Availability

The license terms selected by the author(s) for this preprint version do not permit archiving in PMC. The full text is available from the preprint server.

